# Anti-cancer activity of *Angelica gigas* by increasing immune response and stimulating natural killer and natural killer T cells

**DOI:** 10.1186/s12906-018-2277-7

**Published:** 2018-07-18

**Authors:** Seo Hyun Kim, Sung Won Lee, Hyun Jung Park, Sang Hee Lee, Won Kyun Im, Young Dong Kim, Kyung Hee Kim, Sang Jae Park, Seokmann Hong, Sung Ho Jeon

**Affiliations:** 1 Department of Life Science, Multidisciplinary Genome Institute, Hallym University, Chuncheon, Gangwon 24252 South Korea; 2 Department of Integrative Bioscience and Biotechnology, Institute of Anticancer Medicine Development, Sejong University, Seoul, 05006 South Korea; 3 Medience Co., Ltd., 301, Chuncheon Bioindustry Foundation, Chuncheon, Gangwon 24232 South Korea

**Keywords:** *Angelica gigas*, Immuno-stimulatory polysaccharide fraction, Activation of innate immunity, NK cells, NKT cells, Anti-cancer activity

## Abstract

**Background:**

The polysaccharide component of *Angelica gigas* induces immuno-stimulatory effects on innate immune cells. However, it is unclear whether *A. gigas’* adjuvant activity on the immune system can elicit anti-cancer responses.

**Methods:**

A water-soluble immuno-stimulatory component of *A. gigas* was prepared. How this ISAg modulated the activation of innate immune cells such as dendritic cells (DCs) was examined. ISAg-induced cytotoxic activity via natural killer (NK) and NKT cells was also tested using a tumor-bearing mouse model.

**Results:**

ISAg treatment induced nitric oxide (NO) production and cytokine gene expression involved in innate immune responses. ISAg activated macrophages and DCs to secrete cytokine IL-12, through the TLR4 signaling pathway. IL-12 plays a crucial role in ISAg-mediated NK and NKT cell activation. Thus, the anti-cancer activity of NK and NKT cells induced ISAg-mediated cytotoxicity of B16 melanoma cells in mice.

**Conclusions:**

These results indicated that the natural ingredient, ISAg, has adjuvant activity to induce strong anti-cancer activity of NK and NKT cells in vivo.

## Background

*Angelica gigas* Nakai (i.e., Korean angelica or Dang Gui) is used as a traditional medicinal herb in East Asian countries. Decursin and decursinol angelate are major coumarinic components of the *A. gigas* root, which has anti-cancer [1–3], neuroprotective [4], anti-platelet [5], prevention of obesity [6] and bone-loss [7], and anti-inflammatory [8, 9] properties. Angelan (*A. gigas* peptic polysaccharide) is obtained from water-soluble fraction of *A. gigas* extracts [10]. It has immuno-stimulatory effects through the activation of the innate and adaptive immune systems [11, 12].

Angelan induces splenic lymphocyte proliferation and increases interferon (IFN)-γ production and the immuno-stimulatory cytokine interleukin (IL)-6 during the early stages of treatment [12]. Therefore, macrophages and natural killer (NK) cells in splenocytes might be the main cellular targets directly affected by angelan. Angelan also activates dendritic cell (DC) maturation via the toll-like receptor 4 (TLR4) signaling pathways [11]. Its mechanism of action in lipopolysaccharide (LPS)-induced macrophage activation through the mitogen-activated protein kinase (MAPK) and NF-κB/Rel is well-understood [13]. Angelan also prevents tumor growth and metastasis [14], but the mechanisms via which cells are directly involved in anti-cancer activity are poorly understood. Angelan increases the migration of DCs to lymph nodes; these DCs enhance the anti-tumor activity of the lymphocytes [15]. Release of IL-12 cytokine is one of the effector cell functions of active DCs and macrophages. IL-12 is required for the activation of NK and natural killer T (NKT) cells [16, 17]. NK and NKT cells have major roles in the anti-cancer activity of innate immunity. Infiltration of NK and NKT cells into tumors is closely associated with augmented cytotoxicity against tumor cells, and a much higher survival rate in mice [18, 19].

During the development of natural ingredients for functional food, we separated the water-soluble polysaccharide fraction of *A. gigas* that has immuno-stimulating effects (immuno-stimulatory fraction of *A. gigas*; ISAg). The polysaccharide composition of ISAg is similar to that of angelan [10]. However, ISAg contains a higher fraction of glucose (44.7% of total polysaccharides), which is involved in the TLR4 signaling pathways of macrophages. The objective of this study was to investigate the possible roles of ISAg in induction of the innate immune response and stimulation of the anti-cancer activity of NK and NKT cells.

## Methods

### Preparation of *A. gigas* extract

*Angelica gigas* Nakai root was obtained from Gangwon province, Korea. The voucher specimen (*Y.D. Kim* et al. *TG-20090258*) was deposited at the Herbarium of Hallym University (Chuncheon, Korea). The ISAg was prepared by adding five times greater *v*/v % water to *A. gigas* root and extracting twice at 80 °C for 6 h, and then filtered (pore size, 0.45 μm). The resulting extract was concentrated in vacuo and dissolved in 5 to 8 times 70% ethanol at 55 °C for 2 h with stirring. The ethanol-insoluble precipitates were obtained after centrifugation. The phenol-sulfuric acid method was used to measure the total carbohydrate content of the ISAg [20]. Briefly, 200 μl ISAg was mixed with 1 ml 5% phenol; 5 ml H_2_SO_4_ was then added and mixed well on a vortex mixer. After a 20-min incubation, the color intensity was measured at 490 nm using a Microplate reader (Thermo Fisher Scientific, Waltham, MA, USA). To investigate the constituent sugars, the ISAg was hydrolyzed with H_2_SO_4_ and subjected to anion-exchange high performance liquid chromatography (ICS-5000, Dionex Co., USA) for quantitative analysis.

### Mice and chemical reagents

Wild-type (WT) C57BL/6 (B6), C3H/HeN (TLR4-WT), and C3H/HeJ (TLR4-mutant) mice were obtained from Jung Ang Lab Animal Inc. (Seoul, Korea). IL-12p40 reporter (Yet40) and IL-12p35 knockout (KO) B6 were provided by Dr. R. Locksley (University of California at San Francisco, CA, USA). All mice used in this study were maintained at Hallym University or Sejong University. The animal experiments were approved by the Institutional Animal Care and Use Committee (IACUC) at Hallym University (Hallym 2016–34) and Sejong University (SJ-20160705). All experiments were performed blindly and randomly using age- and sex-matched mice. For sacrifice, mice were euthanized by CO_2_ asphyxiation. The CpG oligodeoxynucleotides (CpG ODN type B 1826) were manufactured by Bioneer (Daejeon, Korea). LPS was obtained from Sigma-Aldrich (St. Louis, MO, USA). Alpha-galactosylceramide (α-GalCer) was obtained from Enzo Life Sciences (Farmingdale, NY, USA).

### Cell culture and cell viability determination

Murine macrophage, RAW264.7 cells were grown in Dulbecco’s modified Eagle’s medium (DMEM; Gibco, Carlsbad, CA, USA) containing 10% fetal bovine serum (FBS, Gibco) supplemented with 2 mM glutamine and 100 units/mL penicillin-streptomycin. Cell viability was measured by using CellTiter 96® AQueous assay kit (Promega, Fitchburg, WI, USA). The cultured cells (5 × 10^4^ cells/well) on 96-well plates were treated with serial dilutions of ISAg for 24 h. MTS tetrazolium was added to the plates and incubated at 37 °C for 1 h. Absorbance was measured at 490 nm using a microplate reader.

### Nitrite assay and enzyme-linked immunosorbent assay (ELISA)

RAW264.7 cells were incubated with LPS (1 μg/mL) or various amounts of ISAg (0.125–2 μg/mL) at 37 °C for 24 h. The amount of nitrite (NO_2_^−^) in the culture supernatant was measured by Griess Reagent System (Promega). The amounts of IL-6, tumor necrosis factor (TNF)-α, and IL-1β secreted to the culture medium were quantified using an ELISA kits (KOMA Biotech, Seoul, Korea).

### Quantitative reverse transcriptase-polymerase chain reaction (qRT-PCR)

Total RNA from RAW264.7 cells was isolated with TRIzol reagent (Invitrogen, Waltham, MA, USA). The relative amount of specific mRNA was assessed by RT-PCR using amfiRivert cDNA Synthesis Platinum Master Mix (GenDEPOT, Baker, TX, USA). Quantification of mRNA was performed using qRT-PCR. The AccuPower® 2X GreenStar™ qPCR Master Mix (Bioneer) and the Exicycler™ 96 PCR system (Bioneer) were used according to the manufacturer’s instructions. The sequences of the sense- and antisense-strand primers used for PCR amplification were inducible nitric oxide synthase (iNOS), 5’-GCTACCACATTGAAGAAGCTGGTG-3′, 5’-CCATAGGAAAAGACTGCACCGAAG-3′; cyclooxygenase-2 (COX-2), 5’-GTCTCTCAATGAGTACCGCAAACG-3′, 5’-CTACCATGGTCTCCCCAAAGATAG-3′; IL-6, 5’-GCCAGAGTCCTTCAGAGAGATACA-3′, 5’-ATTGGATGGTCTTGGTCCTTAGCC-3′; IL-1β, 5’-CCTGTGTAATGAAAGACGGCACAC-3′, 5’-CTTGTGAGGTGCTG ATGTACCAGT-3′; TNF-α, 5’-TCTCATCAGTTCTATGGCCCAGAC-3′, 5’-GGCACCA CTAGTTGGTTGTCTTTG-3′. As a control, glyceraldehyde-3-phosphate dehydrogenase (GAPDH) gene was also amplified using 5’-GACATCAAGAAGGTGGTGAAGCAG-3′, 5’-CCCTGTTGCTGTAGCCGTATTCAT-3′.

### Immunoblot analysis of MAPK

Total cell lysates were extracted using CytoBuster™ Protein Extraction Reagent (Novagen). Equal amounts of protein were separated on 10 to 15% sodium dodecyl sulfate-polyacrylamide gel electrophoresis (SDS-PAGE) gels at 300 mA for 20 min. They were then transferred to Immobilon-P polyvinylidene difluoride (PVDF) membranes (Millipore Sigma, Billerica, MA, USA) using a trans-blot SD Semi-Dry Transfer Cell (Bio-Rad, Hercules, CA, USA). After transfer, the membranes were incubated in Tris buffered saline (TBS), 5% dry milk, and 0.2% Tween 20 for 1 h. They were then further incubated in TBS and 0.2% Tween 20 with specific antibodies at 4 °C overnight. After washing, a horseradish peroxidase (HRP)-labeled secondary antibody was applied and the membranes were incubated for 2 h. Immunoblot detection was performed using Immobilon Western HRP Substrate (Millipore Sigma). The following antibodies from Cell Signaling Technology were used: anti-protein kinase B (PBK/Akt), anti-phospho-Akt (Ser473), anti-c-jun N-terminal kinase (JNK), anti-phospho-JNK, anti-p38, anti-phospho-p38, anti-p44/42 extracellular signal-related kinase (ERK), anti-phospho-p44/42 ERK, and anti-GAPDH.

### Generation of bone marrow-derived DCs (BMDCs)

BMDCs were generated from the bone marrow (BM) cells of Yet40 B6 mice, as previously described [21]. Briefly, BM cells were harvested from femurs and tibiae of mice by flushing with Roswell Park Memorial Institute (RPMI) 1640 medium (Gibco). Red blood cells were removed by adding ACK lysis buffer (0.15 M NH_4_Cl, 10 mM KHCO_3_, and 2 mM EDTA in distilled water), and remaining cells were washed with phosphate buffered saline (PBS) and cultured at a concentration of 1 × 10^6^ cells/well in complete RPMI 1640 medium containing recombinant mouse fms-related tyrosine kinase 3 ligand (Flt3L) (100 ng/ml; R&D Systems, Minneapolis, MN, USA). Fresh cytokine-included culture medium was added on day 5 to generate BMDCs. Five days later, the BMDCs were harvested and stimulated for 16 h with vehicle or ISAg (125 to 2000 ng/ml). The purity of cluster of differentiation (CD)11c^+^ cells of these cultures was > 92%.

### Flow cytometry and intracellular cytokine staining

The following monoclonal antibodies (mAbs) were obtained from BD Biosciences (San Jose, CA, USA): PE-Cy7-, or allophycocyanin (APC)-conjugated anti-CD11c (clone HL3); phycoerythrin (PE)-, or APC-conjugated anti-NK1.1 (clone PK-136); biotin-conjugated anti-TRAIL; PE-Cy7- or APC-conjugated anti-CD3ε (clone 145-2C11); PE-Cy7-conjugated anti-CD11b (clone M1/70); biotin-conjugated anti-CD45 (clone PC61); PE-conjugated anti-IL-12p40 (clone C15.6); biotin-conjugated CD86 (clone GL1); PE-conjugated anti-TNF-α (clone XP6-XT22); PE-conjugated anti-IgG1 (κ isotype control). The following mAbs from Thermo Fisher Scientific were used: PE-conjugated anti-FasL (clone MFL3); APC-conjugated anti-F4/80 (clone BM8); PE-conjugated anti-perforin (clone eBioOMAK-D); PE-conjugated anti-IFN-γ (clone XMG1.2). Flow cytometric data were acquired using a FACSCalibur (Becton Dickinson Inc., San Jose, CA, USA) and were analyzed with FlowJo analysis tool (Tree Star Inc., Ashland, OR, USA). For surface antibody staining, the cells were collected and washed twice with fluorescence-activated cell sorting (FACS) buffer (PBS containing 0.5% bovine serum albumin). The cells were pre-incubated with purified anti-CD16/CD32 mAbs (BD Bioscience, Bedford, MA, USA) on ice for 10 min for blocking non-specific binding to Fc receptors, and were then stained with fluorescence-labeled mAbs. For intracellular staining, splenocytes were incubated with brefeldin A in RPMI 1640 medium (10 μg/ml) at 37 °C for 2 h. Cells were stained for specific surface markers and fixed with 1% paraformaldehyde. After permeabilization with 0.5% saponin (Sigma-Aldrich), cells were stained with the indicated mAbs (PE-conjugated anti-IL-12p40, anti-TNF-α, anti-IFN-γ, and ant-Perforin; PE-conjugated isotype control rat IgG mAbs) for 30 min. More than 5000 events per sample were acquired using the FACSCalibur.

### Cell enrichment using magnetic activated cell sorting (MACS) and cell culture

NK1.1^+^ cells were enriched from total splenocytes isolated from B6 mice using positive selection with anti-APC MACS (Miltenyi Biotec, Bergisch Gladbach, Germany), after staining with APC-conjugated anti-NK1.1 mAb. The cell populations included > 92% NK1.1^+^ cells among all the MACS-purified populations. Splenocytes were prepared as single cell suspensions and cultured in complete RPMI 1640 medium containing 10% FBS supplemented with 5 mM 2-mercaptoethanol, 2 mM L-glutamine, 10 mM HEPES, and 100 units/mL penicillin-streptomycin.

### Cytotoxicity assay

The flow cytometric 7-amino actinomycin D (7-AAD)/carboxyfluorescein succinimidyl ester (CFSE) cytotoxicity assay was used as previously described [22]. NK1.1^+^ cells were isolated as described above and were suspended in complete RPMI 1640 medium. B16 melanoma cells (3 × 10^6^) were stained with CFSE (50 μM) in a 2 ml Hanks’ Balanced Salt Solution at 37 °C for 10 min. NK1.1^+^ cells were incubated with the CFSE-labeled target cells (20,000 cells) at different effector/target (E:T) ratios (27:1, 9:1, 3:1, and 1:1). After a 10-h incubation, the cells were stained with 0.25 μg/ml 7-AAD and were incubated at 37 °C for 10 min. After two washes with PBS containing 1% FBS, cells were resuspended in FACS buffer and their cytotoxicity was evaluated using flow cytometry.

### In vivo ISAg injection procedure

To evaluate the dose-dependent effects of ISAg on innate immune responses, Yet40 B6 mice were given ISAg via oral gavage at doses of 0.5, 1, 2, or 4 mg/mouse, three times per week for 4 weeks. In addition, to measure the time-dependent effects of ISAg on innate immune responses, Yet40 B6 mice were received oral ISAg (4 mg/injection) three times per week for the indicated times (1 to 4 weeks). For experiments to test the involvement of TLR4 signaling in ISAg-mediated innate immune responses, C3H/HeN and C3H/HeJ mice were orally administered ISAg (4 mg/injection) three times per week for 4 weeks. Besides, to investigate whether ISAg-mediated innate immune responses are IL-12 dependent, WT or IL-12p35 KO mice were orally administered ISAg (4 mg/injection) three times per week for 4 weeks. In all the aforementioned experiments, LPS (2 μg/mouse) was used as a positive control to inject mice intraperitoneally (i.p.) once a week for a total of 4 weeks.

### Tumor injection and isolation of tumor-infiltrating leukocytes

To examine in vivo anti-tumor effects of ISAg, Yet40 mice (*n* = 5/group) received subcutaneous (s.c.) injections of 5 × 10^5^ B16 melanoma cells. One week later, these mice were treated orally using oral gavage with either ISAg (4 mg/injection) or PBS three times per week for the following 2 weeks. As a positive control for anti-tumor immune responses, α-GalCer (2 μg) was injected via the intraperitoneal route two times per week starting 7 days after tumor injection, for a total of 2 weeks. On day 21 after tumor injection, groups of mice were euthanized and tumor tissues were excised for immunological analysis. Tumor-infiltrating leukocytes were isolated using the following procedure: small pieces tumor tissues were digested (15 min, 37 °C) using 2.5 mg/ml collagenase type IV (Sigma-Aldrich) and 1 mg/ml DNase I (Promega). After incubation, the digested tissues were dissociated into single-cell suspensions using a gentleMACS Dissociator and C Tubes (Miltenyi). The cell-containing suspension was passed through a 70-μm pore nylon cell strainer (BD Bioscience, Bedford, MA, USA), and put on ice. The tumor-infiltrating leukocytes were harvested from the interface of a 40/70% Percoll (GE Healthcare, Little Chalfont, UK) gradient after centrifugation at 1000 g for 20 min.

### Statistical analysis

Statistical significance was analyzed using the Excel statistical analysis tool (Microsoft, Redmond, WA, USA). The comparison of two groups was performed by the Student’s t-test. Values of *P < 0.05 and **P < 0.01 were considered to indicate a statistically significant result in the Student’s t-test. VassarStats statistical software (http://vassarstats.net/anova2u.html) was used for two-way analysis of variance (ANOVA). Values of ^#^P < 0.05 and ^##^P < 0.01 indicates a statistically significant result in the two-way ANOVA.

## Results

### ISAg stimulated innate immune activity of macrophages

A water-soluble polysaccharide fraction of *A. gigas,* ISAg, was obtained as described in the Methods section. The final product consisted of 63.7% total carbohydrate, 17.4% protein, 0.38% lipid, and inorganic compounds. The maximum content of crude polysaccharide was > 20% of the total extracts. The content of uronic acid, which is a major component of pectin, was 15.1%. The result for the composition of monomeric carbohydrate present in the polysaccharides is presented in Table 1. Glucose was the main polysaccharide component (69.5% of total carbohydrate). The other major sugar constituents were galactose (17.4%) and arabinose (9.0%), Rhamnose, mannose and xylose were also detected as minor components.

**Table 1:** Chemical composition of ISAg and its major monosaccharide components

Chemical composition	Molar ratio (*w*/w %)
Total saccharides	63.7 ± 1.7
Uronic acid	15.1 ± 0.7
Total protein	17.4 ± 0.6
Total lipid	0.4 ± 0.0
Moisture	1.8 ± 0.0
Ash^a^	16.6 ± 0.2
Component Saccharide	Contents (Mole%)^b^
Glucose	69.5 ± 0.6
Galactose	17.4 ± 0.2
Arabinose	9.0 ± 0.2
Rhamnose	2.6 ± 0.2
Mannose	1.2 ± 0.2
Xylose	0.3 ± 0.0

^a^Ash is the non-gaseous, non-liquid residue after complete combustion

^b^Mole% was calculated from the detected total carbohydrate

**Fig. 1:**
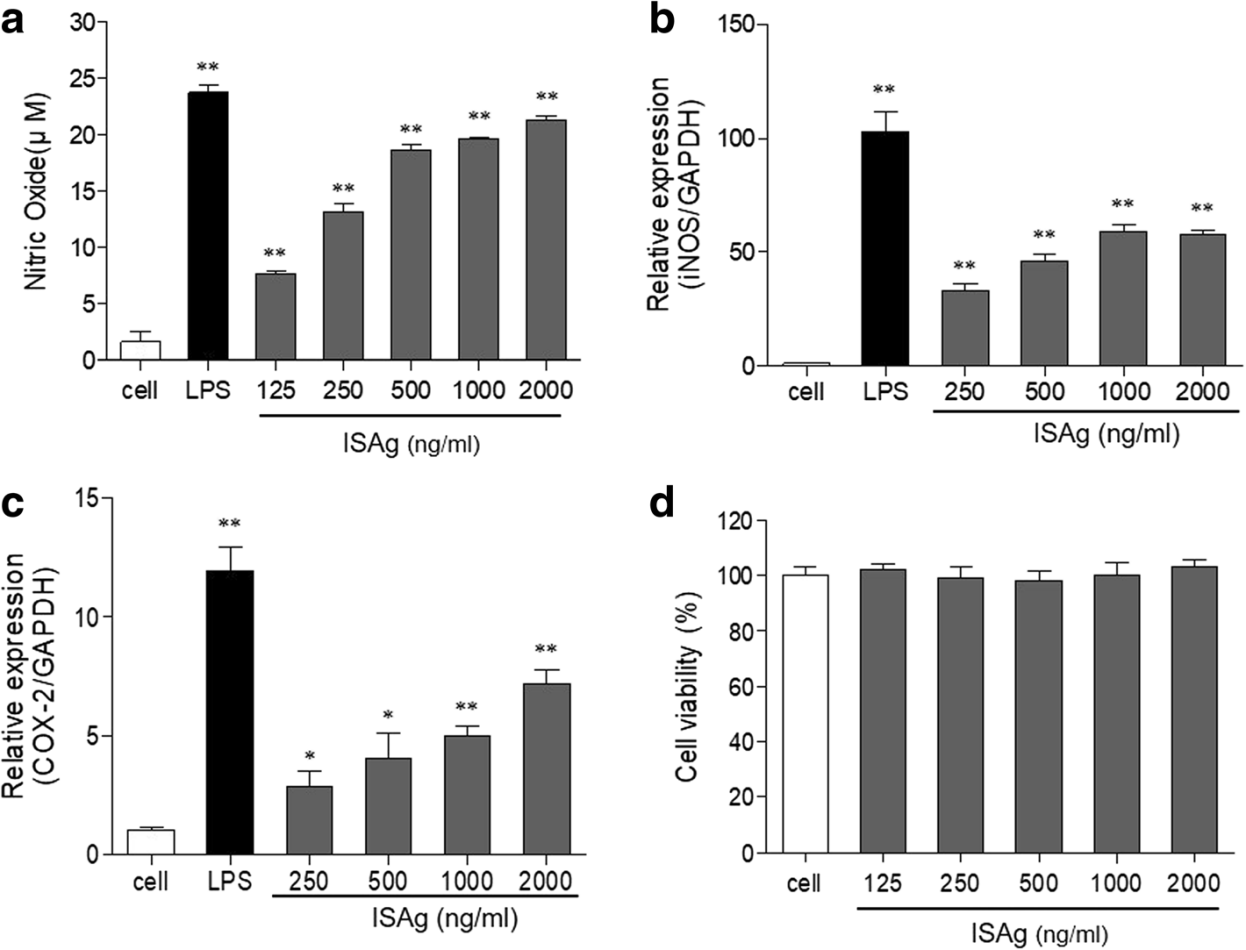
ISAg induced NO production and COX-2 gene expression in murine peritoneal macrophages. **a** RAW264.7 cells were treated with LPS (1 μg/mL) or indicated concentrations of ISAg for 24 h. The amount of NO production was then measured using Griess reagents and an ELISA kit. Gene expression levels of (**b**) iNOS and (**c**) COX-2 relative to GAPDH were analyzed using qRT-PCR. **d** In all experiments, no significant toxic effects of ISAg were detected in the cells. The mean ± standard deviation values are presented (*n* = 3 per group; Student’s t-test; *P < 0.05, **P < 0.01)

To investigate the immuno-stimulatory activity of ISAg, we examined the ISAg-treated mouse macrophage cell line, RAW264.7. ISAg increased NO production of macrophages in a dose-dependent manner, compared with LPS used as a positive control (Fig. 1a). The inducible isoform of nitric oxide synthase (iNOS), which produces NO as an innate immune response defense mechanism, also increased after exposure to ISAg (Fig. 1b). NO stimulates gene expression of COX-2, which converts arachidonic acid to prostaglandin. The transcript level of COX-2 was also increased by the ISAg treatment (Fig. 1c). The high ISAg concentrations (up to 2000 ng/ml) used in all experiments did not induce cytotoxic effects (Fig. 1d).

**Fig. 2:**
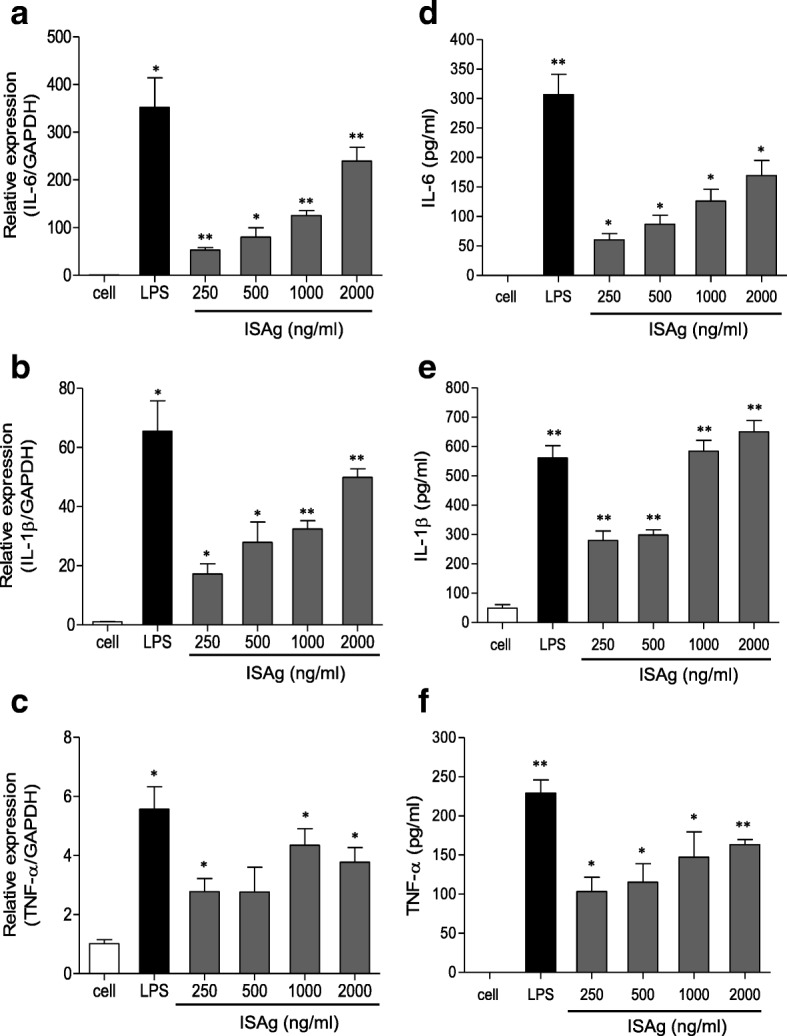
ISAg treatment induced secretion of immuno-stimulatory cytokines from macrophages. **a**–**c** RAW264.7 cells were treated with LPS or indicated concentrations of ISAg. Gene expression levels of (**a**) IL-6, (**b**) IL-1β, and (**c**) TNF-α relative to GAPDH were compared using qRT-PCR. **d**–**f** The levels of each protein secreted in the culture medium were quantified using an ELISA kit. The mean ± standard deviation values are presented (n = 3 per group; Student’s t-test; *P < 0.05, **P < 0.01)

**Fig. 3:**
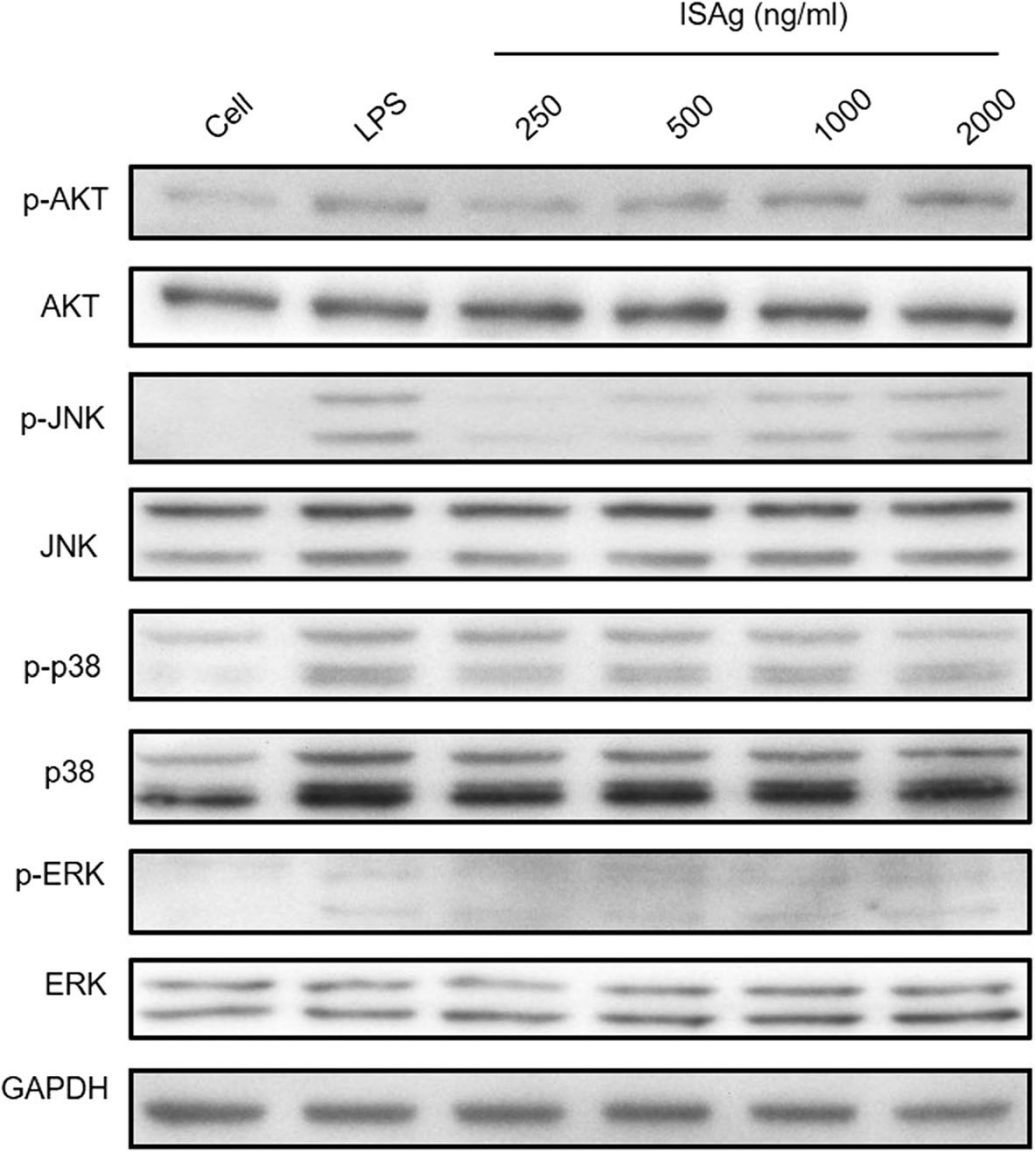
The PBK/Akt and MAPK signaling pathways of macrophages were activated by ISAg treatment. Total cell lysates from RAW264.7 cells treated with LPS or ISAg were separated using SDS-PAGE gel and transferred to PVDF membranes. Phosphorylated-Akt, -JNK, -p38, and -ERK were detected using immunoblot and specific antibodies. GAPDH and the non-phosphorylated form of each protein were used as controls

ISAg also induced macrophages to express the pro-inflammatory cytokine genes IL-6, IL-1β, and TNF-α (Fig. 2). Both the RNA transcripts (Fig. 2a–c) and proteins secreted in the culture medium (Fig. 2d–f) were increased by ISAg treatment. These cytokines activate innate immune responses of pathogen-infected host cells. The immuno-stimulatory effects of ISAg on the production of NO and cytokines might be due to the induction of phosphoinositide 3 kinase (PI3K)/Akt signal transduction pathways and MAPK activity. ISAg significantly induced phosphorylation of Akt and JNK in a dose dependent manner (Fig. 3). Other MAPKs, such as ERK-1/2 and p38, were also phosphorylated after exposure to ISAg. This result was consistent with previously reported results [15].

### ISAg induced IL-12 production in antigen-presenting cells via TLR4 signaling

**Fig. 4:**
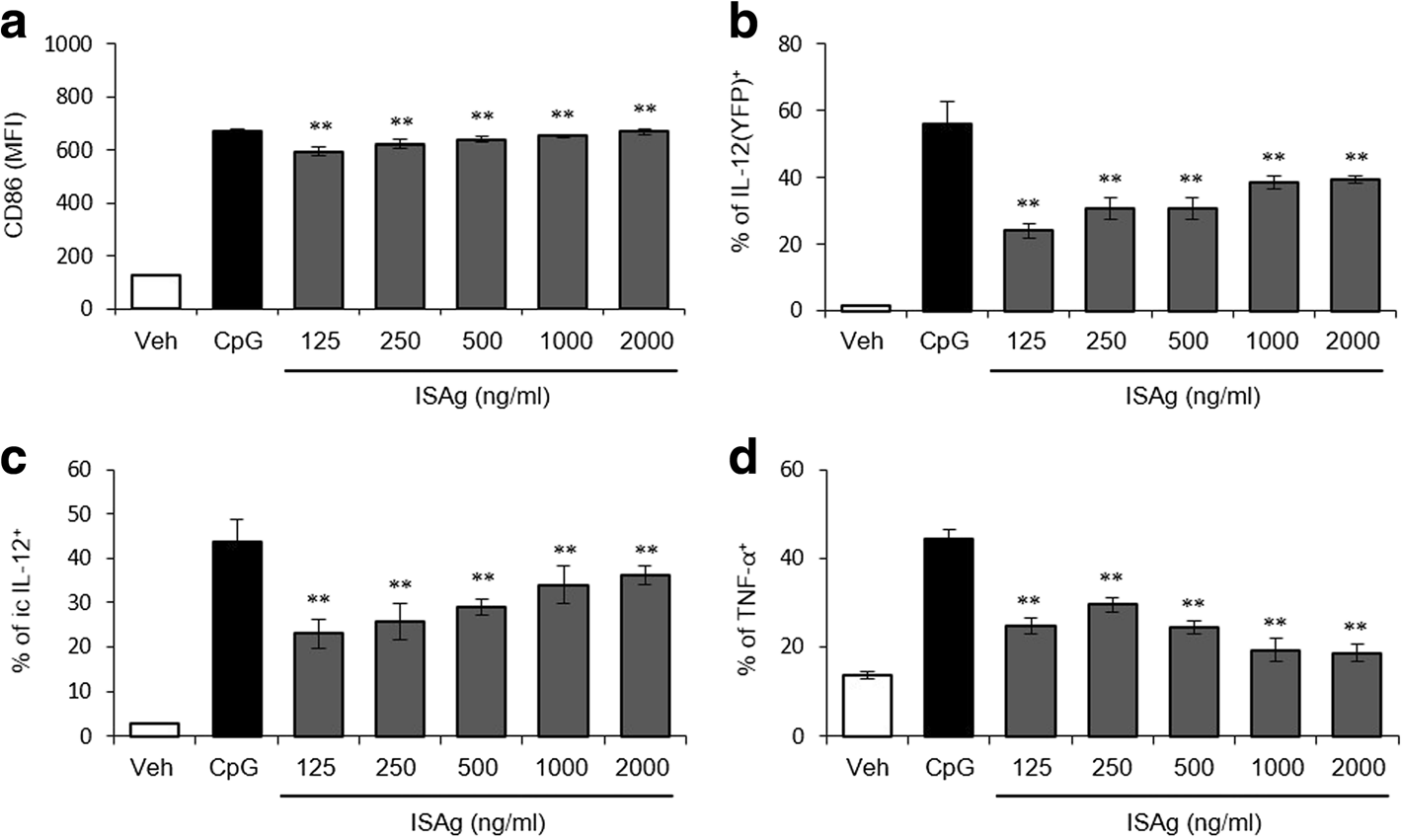
ISAg enhanced production of Th1-type cytokines in BMDCs. Flt3L-cultured BMDCs from Yet40 B6 mice were stimulated for 16 h using a vehicle or the indicated concentration of ISAg. As a positive control, BMDCs were treated with CpG (5 μg/mL). Subsequently, (**a**) the surface expression of CD86 and the production of (**b**) IL-12p40 (YFP), (**c**) intracellular IL-12p40, and (**d**) TNF-α were assessed in CD11c^+^ BMDCs using flow cytometry. The mean ± standard deviation values are presented (*n* = 3 per group; Student’s t-test; **P < 0.01)

Angelan stimulation of DC maturation and migration has anti-cancer effects [11, 15]. Little is known about the details of the cytotoxic process or how it affects the tumor cells. To investigate whether ISAg possessed adjuvant-like ability to stimulate DCs, BMDCs derived from Yet40 B6 mice were prepared and subsequently stimulated with ISAg in vitro. Sixteen hours later, both CD86 expression and cytokine production (IL-12p40 and TNF-α) in BMDCs were examined using flow cytometric analysis. ISAg-treated BMDCs significantly increased the expression of the co-stimulatory molecule CD86 and cytokine production (IL-12p40 and TNF-α) (Fig. 4a–d). Taken together, these results indicated that ISAg had a priming effect during activation of the DCs.

**Fig. 5:**
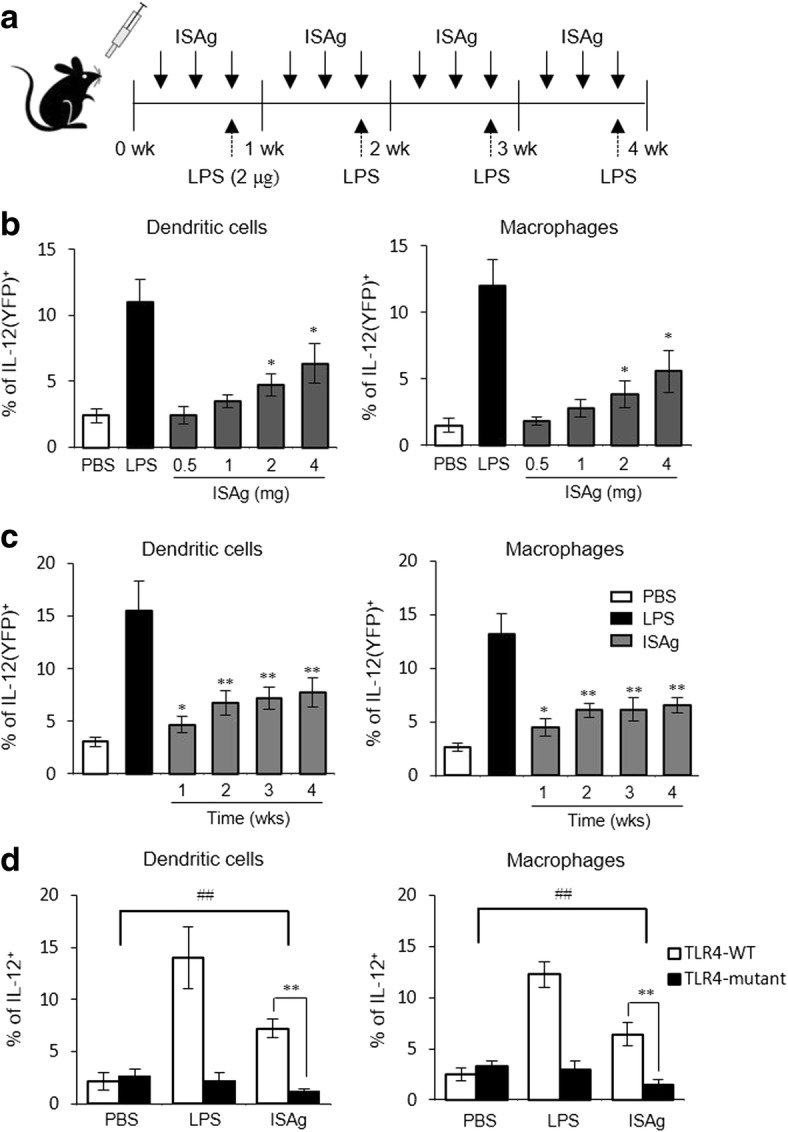
Oral administration of ISAg induced IL-12 production in antigen-presenting cells via TLR4 signaling. **a** Yet40 B6 mice were exposed via the oral route using gavage with ISAg (0.5, 1, 2, or 4 mg/injection) or PBS three times per week for 4 weeks. As a positive control, mice were injected (i.p.) with LPS (2 μg) once per week for 4 weeks. **b** Four weeks later, IL-12p40 (YFP) productions in splenic DCs (CD11c^+^) and macrophages (CD11c^−^CD11b^+^F4/80^+^) were assessed using flow cytometry. **c** Yet40 B6 mice were received oral ISAg (4 mg/injection) three times per week for 1 to 4 weeks. At the indicated times, IL-12p40 (YFP) productions in splenic DCs and macrophages were assessed using flow cytometry. **d** C3H/HeN and C3H/HeJ mice were exposed via the oral route to ISAg (4 mg/injection) or PBS three times per week for 4 weeks. Four weeks later, intracellular IL-12p40 production was analyzed in splenic DCs and macrophages using flow cytometry. The mean ± standard deviation values are presented (*n* = 4 per group; Student’s t-test; *P < 0.05, **P < 0.01). Two-way ANOVA (genotype × treatment) revealed the presence of an interaction between these two factors (^##^*P* < 0.01)

Based on these results, we investigated whether in vivo treatment with ISAg might modulate innate immune cells, including DCs and macrophages. To test this hypothesis, we delivered ISAg into Yet40 mice and then examined the expression of IL-12p40 [yellow fluorescent protein (YFP)] using splenic DCs and macrophages isolated from ISAg-treated mice. We found that in vivo treatment with ISAg caused a statistically significant dose-dependent increase in IL-12 secretion (Fig. 5a, b). Next, to examine the kinetics of ISAg-induced IL-12 production in antigen-presenting cells (APCs), we measured IL-12 production in splenic DCs and macrophages from Yet40 B6 mice fed ISAg (4 mg/injection) three times per week for 1 to 4 weeks. We found that a significant increase in IL-12 secretion by DCs and macrophages was saturated after 2 weeks post-ISAg treatment (Fig. 5c). To further understand how ISAg affects IL-12 production in mouse APCs, we used TLR4-mutant C3H/HeJ and TLR4-sufficient C3H/HeN mice to examine whether TLR4 was responsible for ISAg-mediated IL-12 expression. We found that the enhancing effect of ISAg on IL-12 production was not present in APCs from TLR4-mutant C3H/HeJ mice (Fig. 5d). This result suggested that ISAg-induced IL-12 production in APCs was dependent on the TLR4 signaling pathway.

### ISAg activated NK receptor-expressing innate immune cells via IL-12

**Fig. 6:**
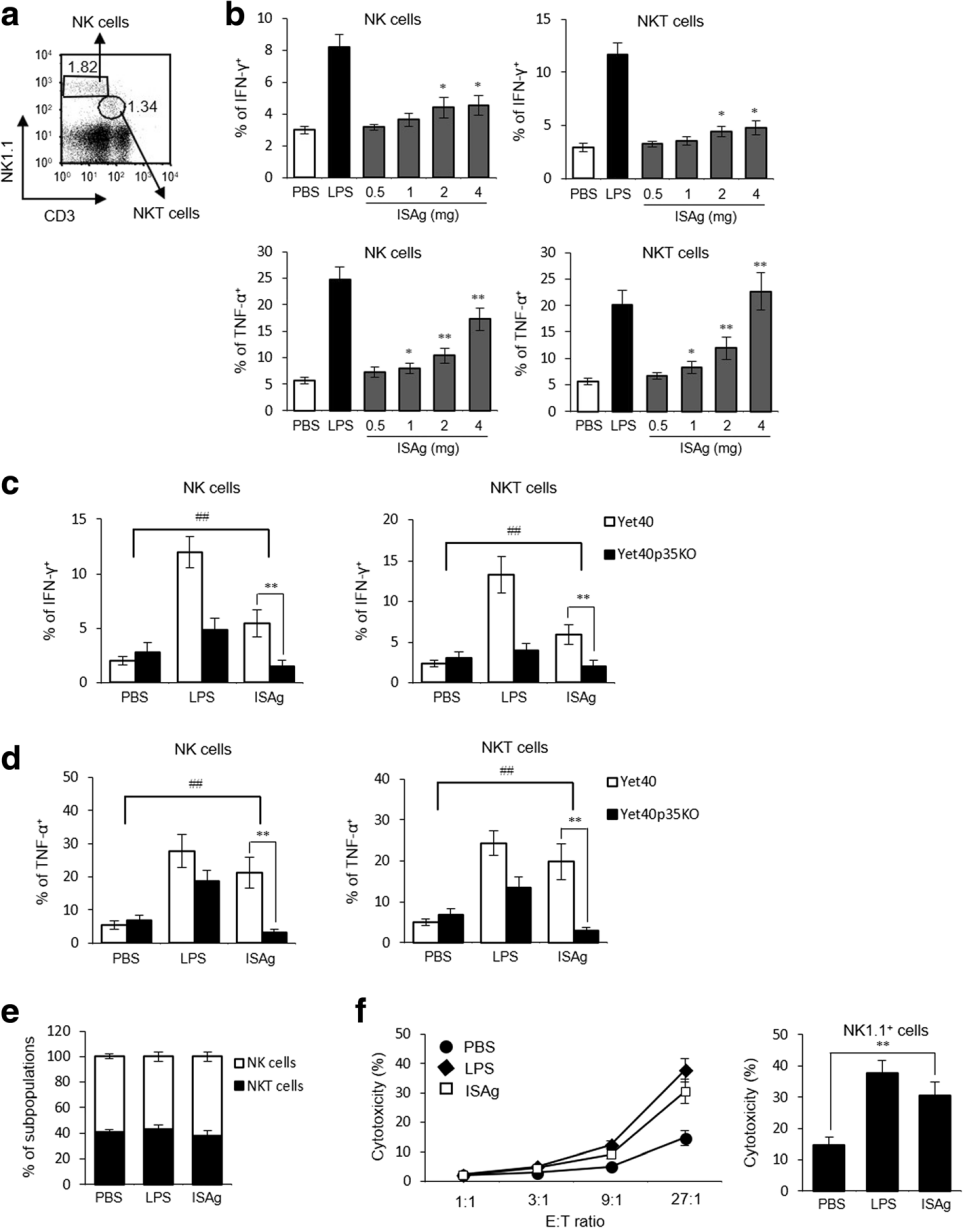
ISAg activated NK receptor-expressing innate immune cells via DC-derived IL-12. **a** The percentages of NK (NK1.1^+^CD3^−^) and NKT cells (NK1.1^+^CD3^+^) among the total splenocytes from Yet40 B6 mice were plotted. **b** Yet40 B6 mice were treated via the oral route with ISAg (0.5, 1, 2, or 4 mg/injection) or PBS three times per week for 4 weeks. As a positive control, mice were injected (i.p.) with LPS (2 μg) once per week for 4 weeks. Intracellular productions of IFN-γ and TNF-α were analyzed in NK and NKT cells. **c**, **d** Yet40 and Yet40p35KO mice were treated via the oral route with ISAg (4 mg/injection) or PBS three times per week for 4 weeks; intracellular production of (**c**) IFN-γ and (**d**) TNF-α were then analyzed in NK and NKT cells. (**e**) NK1.1^+^ cells were isolated from total splenocytes of WT B6 mice treated with ISAg (4 mg/injection), PBS, or LPS [2 μg/injection (i.p.)]. The percentages of NK and NKT cells among the purified NK1.1^+^ cells were measured using flow cytometry. **f** Purified NK1.1^+^ cells were co-cultured with CFSE-labeled B16 tumor cells (2 × 10^4^) at the indicated E:T ratios. After 10 h of co-culture, cytotoxicity was evaluated by calculating the percentage of 7-AAD^+^ (dead) cells, compared with the CFSE^+^ target cells. The mean ± standard deviation values are presented (n = 3 per group; Student’s t-test; *P < 0.05, **P < 0.01). Two-way ANOVA (genotype × treatment) revealed there was an interaction between these two factors (^##^*P* < 0.01)

Our results indicated that ISAg had stimulatory effects on APC activation. However, the effects of ISAg on cytotoxic immune cell (e.g., NK1.1^+^ cells including NK and NKT cells) function have not been studied. To examine these effects, we measured the levels of inflammatory cytokine (IFN-γ and TNF-α) production using splenic NK and NKT cells from Yet40 mice given orally administered ISAg (from 0.5 to 4 mg/mouse). In vivo ISAg treatment showed a statistically significant (*p* < 0.05) increase of NK1.1^+^ cells expressing IFN-γ and TNF-α in Yet40 mice (Fig. 6a, b). We hypothesized that ISAg-associated NK and NKT cell activation is mediated by IL-12. To confirm this, WT or IL-12p35 KO mice were orally administered ISAg and the activation of NK and NKT cells was examined by flow cytometry. The production of inflammatory cytokines in NK and NKT cells of IL-12p35 KO mice was significantly reduced compared to that of WT mice (Fig. 6c, d). These results indicated that IL-12 is required for ISAg-mediated NK and NKT cell activation in vivo. We also examined whether oral administration of ISAg could enhance cytotoxicity and cytokine secretion in NK and NKT cells. NK1.1^+^ cells isolated from PBS- or ISAg-injected WT B6 mice were used as effector cells for the cytotoxicity assay against B16 melanoma cells. We found that in vivo ISAg treatment increased cytotoxicity of NK1.1^+^ cells against the tumor cells (Fig. 6e, f). Based on these results, we propose that NK1.1^+^ cells activated by ISAg treatment can eliminate tumors more efficiently through enhanced effector functions such as cytotoxicity.

### Anti-tumor effects by ISAg were associated with increased cytotoxicity of NK receptor-expressing innate immune cells

**Fig. 7:**
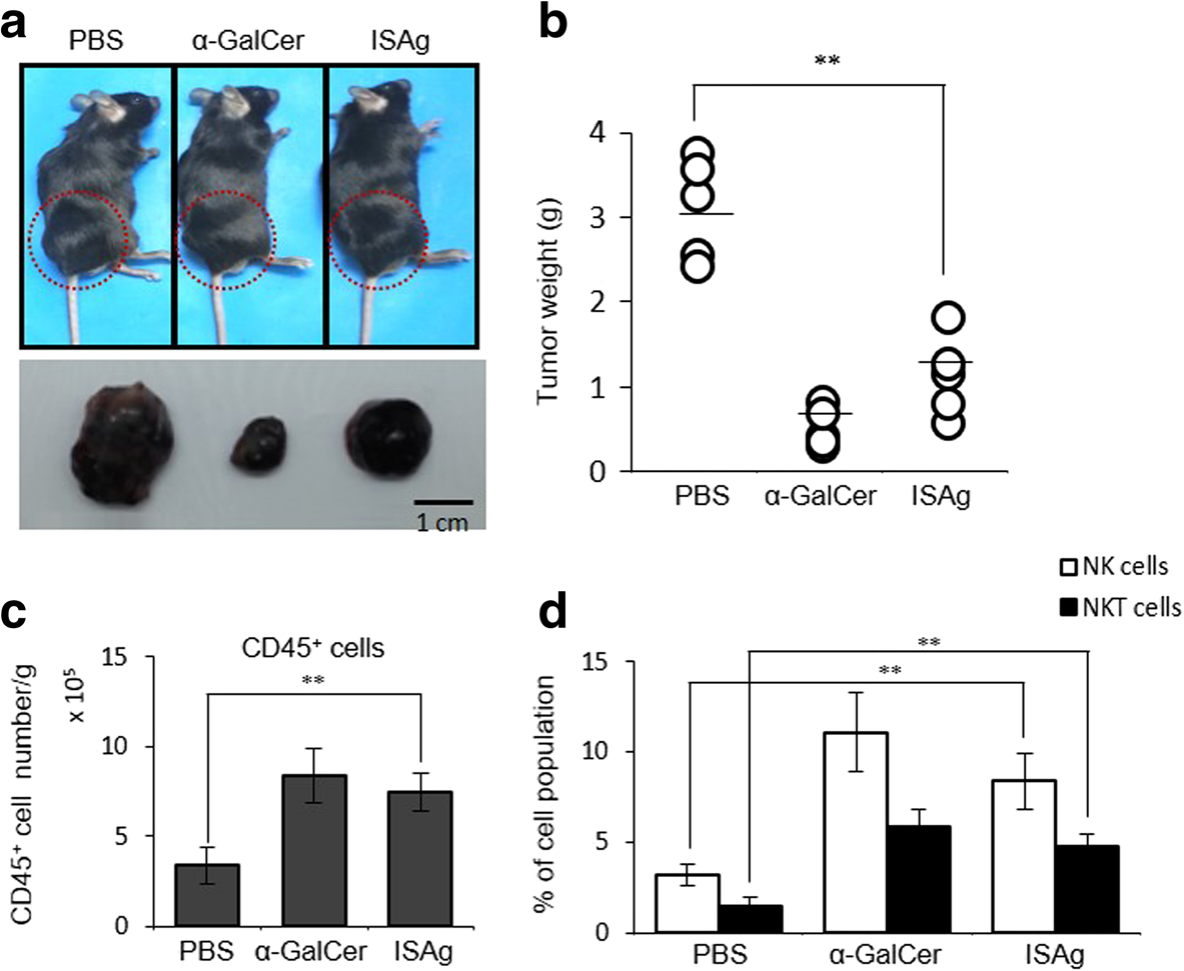
Anti-tumor effects by ISAg were associated with boosted cytotoxicity of NK receptor-expressing innate immune cells. Yet40 B6 mice were treated via the oral route with ISAg (4 mg/injection) or PBS three times per week beginning 1 week after tumor injection and ending at 3 weeks. As a positive control, mice were injected (i.p.) two times per week beginning at 1 week after tumor injection until 3 weeks with α-GalCer (2 μg) dissolved in PBS. The mice were euthanized at day 21 after B16 tumor cell injection. The spleen and tumor tissues were excised from each mouse. **a** Representative photographs of a mouse from each treatment, 21 days after tumor injection (top row). Photographs of the tumors from each of the mice in the top row (bottom row). **b** The weights of the tumors from individual mice are presented mean ± standard deviation values (*n* = 5 per group; Student’s t-test; **P < 0.01). **c**, **d** On day 21 after injection, mononuclear cells were isolated from the tumors of mice in the indicated treatment groups using a Percoll gradient. **c** The absolute numbers of CD45^+^ cells per gram of tumor tissue were assessed using flow cytometry. **d** The frequencies of the NK and NKT cells among the CD45^+^ cells from the tumors were evaluated using flow cytometric analysis

**Fig. 8:**
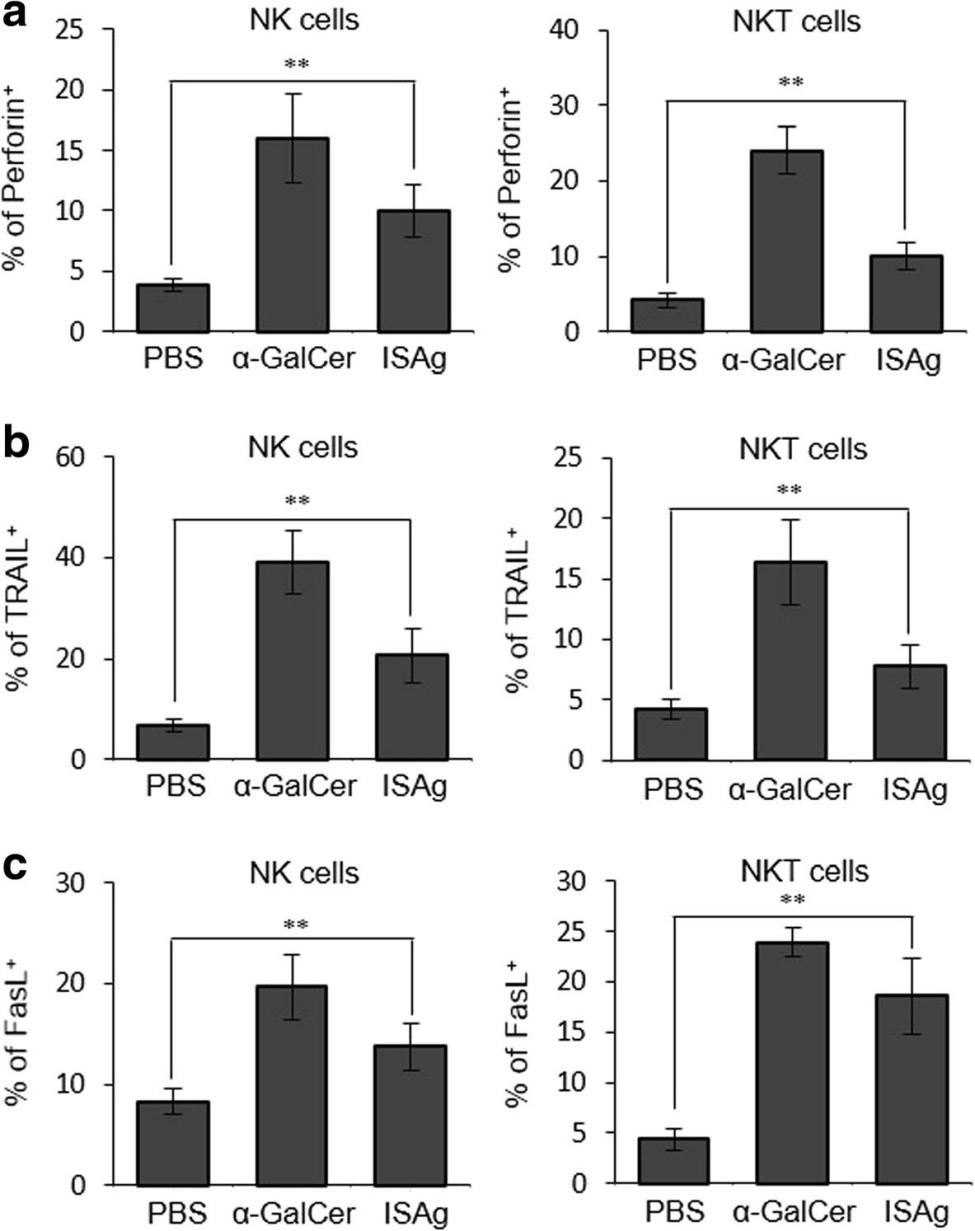
ISAg induced expression of cytotoxic molecules in NK and NKT cells. The preparation of splenic NK1.1^+^ cells is described in the Fig. 7 legend. Expressions of (**a**) perforin, (**b**) TRAIL, and (**c**) FasL by splenic NK and NKT cells were evaluated using flow cytometry. The mean ± standard deviation values are presented (n = 5 per group; Student’s t-test; **P < 0.01)

Based on the results of the in vitro experiments that examined activation of NK receptor-expressing innate immune cells via IL-12, we hypothesized that in vivo ISAg treatment can inhibit tumor growth in mice. To examine in vivo anti-tumor activity of ISAg, Yet40 mice were inoculated (s.c.) with B16 melanoma. One week later ISAg was orally injected to these mice. ISAg-injected mice had a significant decrease in tumor growth, compared with PBS-injected mice (Fig. 7a, b). To examine whether the increased accumulation of NK and NKT cells was correlated with a decrease in tumor mass, we compared the numbers of NK and NKT cells infiltrated into the tumors between PBS- and ISAg-injected mice. There was a marked increase in accumulation of NK and NKT cells in B16 tumors after ISAg treatment, compared with the PBS-treated mice (Fig. 7c, d). Next, we examined whether there was any change in the expression of cytotoxic molecules such as perforin, TRAIL, or FasL, following ISAg administration. All these molecules were significantly increased in NK and NKT cells after ISAg treatment in vivo (Fig. 8a–c). These results indicated that the cytotoxicity of ISAg treatment against B16 tumor cells in mice was mainly due to the anti-cancer activity of NK and NKT cells.

## Discussion

Natural compounds obtained from the same plant using different kinds of solvents (usually water-soluble versus water-insoluble) often have opposite effects on human and experimental animal physiology [8, 11]. However, contradictory (immuno-stimulatory or inhibitory) regulation of immune responses induced by these extracts can achieve the same goal of anti-cancer activity. Decursin induces direct cytotoxic effects on tumor cells through cell cycle arrest [23] or activation of the protein kinase C and reactive oxygen species signaling pathways [24]. Our study found that the polysaccharide component of *A. gigas* induced the death of target tumor cells through activation of the immune system.

Depending on the microenvironment, macrophages can be polarized into either an M1 phenotype with anti-tumor properties or an alternative M2 phenotype with pro-tumor properties [25]. Components of *A. gigas* extract can induce distinct immune responses that are pro-inflammatory or anti-inflammatory [11, 26]; our study revealed that ISAg treatment preferentially differentiated macrophages toward the M1 phenotype (producer of TNF-α and IL-12), but not the M2 phenotype (producer of IL-10). These results indicated that the M1 polarization of macrophages by ISAg treatment is a mechanism that elicits an optimal anti-tumor response. Angelan increases the maturation of DCs via TLR4 [11], but the signaling pathway of ISAg is unclear because the major components of ISAg polysaccharide are quite different. In conclusion, we found that macrophages and DCs were activated by ISAg through TLR4 signaling pathway and subsequently secreted IL-12 cytokine.

NK and NKT cells are activated via ISAg-induced IL-12, which results in increased levels of cytolytic molecules including perforin, TRAIL, and FasL. Thus, TLR4- and IL-12-dependent NK and NKT cell activation by ISAg ultimately results in enhanced cytotoxicity against tumor cells. Previous studies found that angelan induces proliferation of splenic B lymphocytes [12], but T-cells can be indirectly activated [15]. Regulatory T (Treg) cells are required for suppression of the innate anti-tumor immunity of NK and NKT cells [27, 28]. Inversely, activated NK and NKT cells can inhibit the development of Treg cells via IFN-γ production [29, 30]. Consistent with the results of previous studies, our results indicated that ISAg increased IFN-γ production by NK and NKT cells, resulting in a significant decrease in the Treg population within the tumor tissue (data not shown). This result suggested that enhanced anti-tumor function by ISAg treatment might be related to the suppression of Treg cells resulting from activation of NK and NKT cells. Moreover, our previous results indicated that anti-tumor effects of human T helper type 1 (Th1)-type cytokine (IL-32γ) are attributed to induction of DC maturation followed by consequent enhancement of NK and NKT cell cytotoxicity [31] via DC-derived IL-12 manner [16]. Therefore, it will be of interest to apply ISAg as an immune adjuvant or functional food ingredient to boost anti-cancer immune responses.

## Conclusions

This study suggests the following scenario in which ISAg can induce more effective anti-tumor immunity. First, oral administration of ISAg initiates phenotypic changes in innate immune cells such as macrophages and DCs. These results lead to polarization of macrophages to the M1 phenotype as well as DCs to the immunostimulatory phenotype. Second, such ISAg-induced IL-12 subsequently activates NK and NKT cells in order to produce cytotoxic cytokines, especially IFN-γ and TNF-α. Consequently, in vivo ISAg treatment could effectively elicit anti-tumor immune responses. Taken together, our findings strongly suggest that ISAg is an excellent natural product that helps boost the anti-cancer immune responses.

## Abbreviations

7-ADD: 7-amino actinomycin D
ACK: Ammonium-chloride-potassium
APC: Allophycocyanin
APCs: Antigen-presenting cells
B6: C57BL/6
BMDCs: Bone marrow-derived dendritic cells
CD: Cluster of differentiation
CFSE: Carboxyfluorescein succinimidyl ester
COX-2: Cyclooxygenase-2
DCs: Dendritic cells
DMEM: Dulbecco’s modified Eagle’s medium
ELISA: Enzyme linked immunoabsorbance assay
ERK: Extracellular signal-related kinase
FACS: Fluorescence-activated cell sorting
FBS: Fetal bovine serum
Flt3L: Fms-related tyrosine kinase 3 ligand
GAPDH: Glyceraldehyde-3-phosphate dehydrogenase
HRP: Horseradish peroxidase
i.p.: Intraperitoneally
IFN: Interferon
IL: Interleukin
iNOS: Inducible nitric oxide synthase
ISAg: Immuno-Stimulatory fraction of *A. gigas*
JNK: c-jun N-terminal kinase
LPS: Lipopolysaccharide
mAbs: Monoclonal antibodies
MACS: Magnetic activated cell sorting
MAPK: Mitogen-activated protein kinase
NK: Natural killer
NKT: Natural killer T
NO: Nitric oxide
PBK/Akt: Protein kinase B
PBS: Phosphate buffered saline
PE: Phycoerythrin
PI3K: Phosphoinositide 3 kinase
PVDF: Polyvinylidene difluoride
RPMI: Roswell Park Memorial Institute
s.c.: Subcutaneous
SDS-PAGE: Sodium dodecyl sulfate-polyacrylamide gel electrophoresis
TBS: Tris buffered saline
Th1: T helper type 1
TLR4: Toll-like receptor 4
TNF: Tumor necrosis factor
Treg: Regulatory T
YFP: Yellow fluorescent protein
α-GalCer: Alpha-galactosylceramide
